# Childhood Adversity and Health in Emerging Adulthood

**DOI:** 10.1001/jamanetworkopen.2026.35834

**Published:** 2026-09-24

**Authors:** Jeff R. Temple, Elizabeth Baumler, Leila Wood, Leslie Taylor

**Affiliations:** 1 Univeristy of Texas Health, School of Behavioral Health Sciences, Houston

## Abstract

**Importance:**

Adverse childhood experiences (ACEs) are robust estimators of poor health across the lifespan, yet most research relies on retrospective reports from adult samples and conventional ACE measures that exclude community-level adversity. Focusing on emerging adulthood—a developmental period when adversity may exert increased lifetime associations—provides a clearer view of near-term impacts and a critical window for intervention.

**Objectives:**

To examine associations between conventional and community-level ACEs and mental health, substance use, interpersonal violence, and educational outcomes in emerging adulthood, and to assess the relative contributions of ACE domains to key outcomes.

**Design, Setting, and Participants:**

Longitudinal cohort study using data collected in waves 5 (March to August 2023) and 6 (February to August 2024) of a population-based sample recruited from urban and suburban schools in the Southern US. Of 2768 participants, 1850 with complete data were included in the present analyses.

**Exposures:**

Conventional and community-level ACEs assessed at wave 6, categorized as 0, 1 to 3, 4 to 5, or 6 or more exposures.

**Main Outcomes and Measures:**

Depression, posttraumatic stress disorder, suicidality, substance use, dating violence (DV), sexual assault, and academic mobility.

**Results:**

Among the 1850 participants (mean [SD] age at wave 6, approximately 19 [0.6 years; 1395 [50.4%] female; 389 [14.1%] Asian, 709 [25.6%] Black, 1025 [37.0%] Hispanic, 200 [7.2%] White, and 445 [16.1%] other race and ethnicity), higher ACE exposure showed graded associations with nearly all outcomes. Compared with 0 ACEs, those with 4 to 5 and 6 or more ACEs had higher rates of depression (70 [21.0%] individuals with 0 ACEs vs 165 [46.5%] with 4 to 5 ACEs and 141 [56.9%] with 6 or more ACEs), PTSD (7 [2.1%] with 0 ACEs vs 54 [15.2%] with 4 to 5 ACEs and 77 [30.9%]), suicidal ideation (20 [6.0%] with 0 ACEs vs 98 [27.8%] with 4 to 5 ACEs and 103 [41.4%] with 6 or more ACEs), illicit substance use (ie, speed, hallucinogens, heroin, or ecstasy; 1 [0.3%] with 0 ACEs vs 22 [6.3%] with 4 to 5 ACEs and 27 [10.8%] with 6 or more ACEs), and interpersonal violence (eg, experiencing physical DV, 9 [2.7%] with 0 ACEs vs 30 [8.5%] with 4 to 5 ACEs and 37 [14.9%] with 6 or more ACEs). Academic achievement and college attendance decreased with increasing ACE exposure. Ten of 14 ACE domains were independently associated with key outcomes and 11 with experiencing DV. Emotional abuse, sexual abuse, witnessing domestic violence, and discrimination showed the most consistent associations.

**Conclusions and Relevance:**

In this cohort study of emerging adults, both conventional and community-level ACEs were associated with poorer mental health, interpersonal violence, substance use, and reduced academic mobility. These findings highlighted emerging adulthood as a critical period for intervention and suggested that, beyond cumulative exposure, specific forms of adversity were more consistently associated with adverse outcomes than others.

## Key Points

**Question:**

How are conventional and community-level adverse childhood experiences associated with mental health, substance use, interpersonal violence, and educational outcomes during emerging adulthood?

**Findings:**

In this cohort study of 1850 emerging adults, higher adverse childhood experience exposure was associated with elevated rates of depression, posttraumatic stress disorder, suicidality, interpersonal violence, lower academic achievement, and use of narcotics, opioids, and other illicit substances. Both adverse childhood experience types contributed to risk; however, specific domains—particularly emotional abuse, sexual abuse, witnessing domestic violence, and discrimination—demonstrated more consistent associations.

**Meaning:**

In this study, adversity across households and communities showed graded associations with functioning, underscoring the need for prevention strategies addressing cumulative burden and high-impact adversities, including structural conditions.

## Teaser

This cohort study examines associations between adverse childhood experiences and mental health, substance use, interpersonal violence, and educational outcomes in emerging adulthood.

## Introduction

Adverse childhood experiences (ACEs) are well-established risk factors for poor health across the lifespan.^1,2,3,4,5^ Although the original ACE framework focused on abuse and household dysfunction,^1^ its measurement was shaped largely by White, insured, middle-class adults.^1,2,6^ More recent work has expanded ACEs to include community-level adversities, such as neighborhood violence, discrimination, and unsafe neighborhood conditions whose impacts fall disproportionately on racially and socioeconomically marginalized individuals.^6,7,8^

Despite these conceptual advances, 3 major gaps remain. First, most ACE studies still rely on retrospective reports from middle-aged adults,^9,10,11,12^ a practice that introduces recall bias and obscures the near-term developmental consequences of early adversity. Second, relatively little work has examined ACEs in ethnically diverse emerging adults (age, 18-25 years), even though adversity’s associations with mental, behavioral, and educational outcomes are often most pronounced during this developmental period.^13,14^ Finally, the relative impact of individual ACEs is often lost when summary scores are used exclusively for analysis.^15^

To address these gaps, we examined conventional and community-level ACEs in a racially and ethnically diverse cohort of emerging adults, focusing on mental health, substance use, interpersonal violence, and academic mobility. We also assessed whether specific ACE domains were more associated with key mental health and interpersonal violence outcomes.

## Method

### Study Design and Participants

We used waves 5 (2023) and 6 (2024) of an ongoing longitudinal cohort established in 2018 as part of a cluster-randomized trial of a school-based healthy relationships intervention. Students in participating classrooms at middle schools in 2 urban and suburban southeast Texas districts were eligible. Written parental consent and student assent were obtained at baseline; no additional inclusion or exclusion criteria were applied. Participants have been followed annually. Race and ethnicity were self-reported by participants and were included because prior research has demonstrated differences in both ACE exposure and health outcomes across racial and ethnic groups. Retention was high, with 76.0% (n = 2104) participating at wave 5 (mean age = 18 years) and 73.4% (n = 2033) at wave 6 (mean age = 19 years). The analytic sample included 1850 participants with follow-up at waves 5 and 6. We used a complete case analysis approach on relevant variables at both waves. The study was approved by the UTHealth Houston institutional review board. This study used Strengthening the Reporting of Observational Studies in Epidemiology (STROBE) reporting guidelines.

### Exposure Measure

At wave 6, when participants were at least 18 years old, we used the 21-item Philadelphia ACEs survey to assess conventional (eg, child maltreatment) and community-level (eg, neighborhood safety) forms of childhood trauma that participants reported retrospectively.^6^ Using the prescribed scale measurement coding,^6^ individual survey items were grouped into 14 ACE domains representing distinct types of adversity (eg, emotional abuse as a child, physical abuse as a child, experiencing discrimination, exposure to adverse neighborhood experiences; see eTable 1 in Supplement 1). The total number of ACEs was calculated for each participant by summing the number of ACEs endorsed. Sensitivity analyses were then conducted to create categories of 0, 1 to 3, 4 to 5, and 6 or more. Domain-level indicators were used for each of the 14 domains to examine cooccurrence and to estimate independent and associations with selected outcomes.

### Outcome Measures

At wave 5, we assessed depression with the Center for Epidemiologic Studies Depression-10^16^; scores of 10 or higher were considered positive.^16,17^ Among participants reporting a traumatic event (n = 447), we assessed PTSD with the Primary Care–posttraumatic stress disorder–5^18^; endorsement of at least 3 or more items was considered positive. We created 2 items to assess individuals with suicidal ideation (ie, “Have you thought about suicide in the past year?”; yes or no) and attempts (ie, “Have you attempted suicide in the past year?”; yes, no, or I have attempted suicide, but it was more than a year ago). This dichotomous outcome was coded yes for any suicide attempt.

At wave 5, we assessed past-month binge drinking (≥4 drinks on 1 occasion for females; ≥5 for males), as well as past year use (yes or no) of marijuana and illicit substances. For the latter, we considered participants who endorsed any past year use of speed, hallucinogens, heroin, or ecstasy as positive for use of narcotics, opioids, and other illicit substances.

At wave 5, we used the Conflict in Adolescent Dating Relationships Inventory^19^ to assess past-year experiences of dating violence (DV) and perpetration of DV, including physical, sexual, psychological, and threatening abuse. Any endorsement was coded positive, except psychological abuse, which required endorsement of at least 4 or more items.^20,21^ We used a modified version of the Sexual Experiences Survey^22^ to assess lifetime history of sexual assault. Additionally, we asked participants (yes or no) if they had been in a physical fight in the last year. At wave 5, we asked participants about their academic achievement in high school (yes or no to an A or B average) and whether (yes or no) they graduated, as well as whether they are attending a 4-year college (yes or no).

### Statistical Methods

We calculated ACE sum scores and, based on sensitivity analyses and prior literature, categorized exposure as 0, 1 to 3, 4 to 5, or at least 6 or more ACEs. We estimated prevalence and 95% CIs overall and by ACE category. We used multilevel logistic regression models to estimate associations between ACE category and each outcome, adjusting for sex, race and ethnicity, perceived income, sexual orientation, and treatment group, with clustering by school. Odds ratios represent the association between ACE exposure and each outcome relative to participants with no ACE exposure. Although ACEs were assessed retrospectively at wave 6 (February to August 2024), participants were asked to report experiences that occurred before age 18 years. Most outcomes were assessed at wave 5 (March to August 2023) and referred to the preceding year or current symptoms. Thus, despite the later administration of the ACE measure, the reported exposures generally preceded the outcomes conceptually, although some overlap around age 18 was possible.

We estimated the prevalence and 95% CIs for each of the 14 ACEs domains. We then calculated the bivariate correlations across each of the 14 ACE domains to assess cooccurrence (eTable 2 in Supplement 1). To examine domain-specific ACEs associations, we fit multilevel logistic regression models adjusted for sex, race and ethnicity, sexual orientation, income, and treatment group. Given the large number of potential comparisons, ACE domain analyses were limited a priori to depression, PTSD, experiences of DV, and perpetration of DV. Model 1 estimated associations for each domain separately; model 2 included all 14 domains simultaneously to estimate relative associations. Statistical significance was 2-sided and determined using an a priori hypothesis test set at *P* < .05.

## Results

The analytic sample consisted of 1850 participants (mean [SD] age, 19 [0.6] years), of whom 1002 (54.2%) were female, 309 (16.7%) were Asian, 425 (23.0%) were Black, 674 (36.4%) were Hispanic, 110 (5.9%) were White, and 332 (17.9%) identified as another race and ethnicity (Table 1). As shown in Table 2, rates of mental health, substance use, and interpersonal violence (eg, DV, peer fight, and sexual assault) outcomes increased with greater ACE exposure. Compared with 0 ACEs, those with 4 to 5 and 6 or more ACEs had higher rates of depression (70 [21.0%] individuals with 0 ACEs vs 165 [46.5%] with 4 to 5 ACEs and 141 [56.9%] with 6 or more ACEs), PTSD (7 [2.1%] with 0 ACEs vs 54 [15.2%] with 4 to 5 ACEs and 77 [30.9%]), suicidal ideation (20 [6.0%] with 0 ACEs vs 98 [27.8%] with 4 to 5 ACEs and 103 [41.4%] with 6 or more ACEs), illicit substance use (ie, speed, hallucinogens, heroin, or ecstasy; 1 [0.3%] with 0 ACEs vs 22 [6.3%] with 4 to 5 ACEs and 27 [10.8%] with 6 or more ACEs), and interpersonal violence (eg, experiencing physical DV, 9 [2.7%] with 0 ACEs vs 30 [8.5%] with 4 to 5 ACEs and 37 [14.9%] with 6 or more ACEs). Similarly, ACE exposure was inversely associated with all 3 academic mobility outcomes. As shown in Table 3, exposure to 4 to 5 or 6 or more ACEs was associated with significantly higher odds of all examined mental health, substance use, and interpersonal violence outcomes compared with no ACEs. Some outcomes, including depression, PTSD, suicidality, psychological DV, sexual assault, and marijuana and illicit substance use, showed elevated risk even for those with exposure to 1 to 3 ACEs. College enrollment was inversely associated with the highest level of ACEs exposure (6 or greater). Furthermore, high school academic achievement was inversely associated with all categories of exposure to ACEs.

**Table 1.** Participant Characteristics

Characteristic	Participants, No. (%)	
	Baseline cohort (n = 2768)	Analytical sample (n = 1850)
Sex		
Female	1395 (50.4)	1002 (54.2)
Male	1373 (49.6)	848 (45.8)
Race and ethnicity^a^		
Asian	389 (14.1)	309 (16.7)
Black	709 (25.6)	425 (23.0)
Hispanic	1025 (37.0)	674 (36.4)
White	200 (7.2)	110 (5.9)
Other^b^	445 (16.1)	332 (17.9)

^a^ Race and ethnicity categories are mutually exclusive. Participants self-reported their race and ethnicity.

^b^ Includes participants who endorsed American Indian or Alaska Native, other, or multiple.

**Table 2.** Outcome Descriptives Overall and Stratified by ACE Category

Outcome	No. (%) [95% CI]^a^				
	Overall (N = 1850)^b^	No ACEs (n = 337)	1 to 3ACEs (n = 878)	4 to 5 ACEs (n = 355)	6 or more ACEs (n = 253)
Mental health					
Depression	695 (37.9) [35.7- 40.2]	70 (21.0) [16.6 to 25.4]	313 (36.0) [32.8-39.2]	165 (46.5) [41.3-51.7]	141 (56.9) [50.7-63.1]
PTSD	210 (11.4) [10.0-12.9]	7 (2.1) [0.6-3.6]	69 (7.5) [6.1-9.7]	54 (15.2) [11.5-19.0]	77 (30.9) [25.1-36.7]
Suicide ideation	390 (21.3) [19.4-23.2]	20 (6.0) [3.4-8.5]	166 (19.1) [16.5-21.7]	98 (27.8) [23.1-32.6]	103 (41.4) [35.2-47.5]
Suicide attempt^c^	278 (15.1) [13.5-16.8]	15 (4.5) [2.3-6.7]	95 (10.9) [8.8-13.0]	72 (20.4) [16.2-24.6]	93 (37.2) [31.2-43.2]
Experiencing DV by type					
Physical	109 (6.0) [4.9-7.0]	9 (2.7) [1.0-4.5]	32 (3.7) [2.4-4.9]	30 (8.5) [5.6-11.5]	37 (14.9) [10.4-19.3]
Sexual	81 (4.4) [3.4-5.3]	4 (1.2) [0.03-2.4]	26 (3.0) [1.9-4.1]	21 (5.9) [3.5-8.4]	29 (11.6) [7.6-15.6]
Threatening	122 (6.7) [5.5-7.8]	8 (2.4) [0.8-4.1]	43 (4.9) [3.5-6.4]	32 (9.1) [6.1-12.1]	39 (15.6) [11.1-20.1]
Psychological	306 (16.6) [14.9-18.3]	24 (7.1) [4.4-9.9]	123 (14.0) [11.7-16.4]	87 (24.5) [20.0-29.0]	71 (28.3) [22.7-33.9]
DV perpetration by type					
Physical	96 (5.2) [4.2-6.3]	5 (1.5) [0.2-2.8]	31 (3.6) [2.3-4.8]	25 (7.1) [4.4-9.8]	35 (14.1) [9.7-18.4]
Sexual	31 (1.7) [1.1-2.3]	0 (0.0) [NA]	12 (1.4) [0.1-2.2]	6 (1.7) [0.03-3.0]	13 (5.2) [2.4-8.0]
Threatening	101 (5.5) [4.5-6.6]	5 (1.5) [0.2-2.8]	29 (3.3) [2.1-4.5]	25 (7.1) [4.4-9.8]	40 (16.0) [11.1-20.6]
Psychological	272 (14.7) [13.1-16.4]	18 (5.4) [2.9-7.8]	101 (11.5) [9.4-13.7]	74 (20.8) [16.6-25.1]	76 (30.3) [24.6-36.0]
Other violence					
Sexual assault^c^	182 (9.9) [8.6-11.3]	8 (2.4) [0.8-4.1]	58 (6.6) [5.0-8.3]	44 (12.5) [9.0-16.0]	69 (27.6) [22.0-33.2]
Physical fight	140 (7.6) [6.4-8.9]	8 (2.4) [0.8-4.1]	40 (4.6) [3.2-6.0]	43 (12.1) [8.7-15.5]	48 (19.2) [14.3-24.1]
Substance use					
Binge drinking	194 (10.7) [9.2-12.1]	20 (6.0) [3.5-8.6]	78 (9.0) [7.1-10.9]	51 (14.5) [10.8-18.2]	42 (17.1) [12.3-21.8]
Marijuana use	406 (22.1) [20.2-24.0]	37 (11.1) [7.7-14.5]	156 (17.8) [15.3-20.4]	107 (30.2) [24.4-35.0]	98 (39.2) [33.1-45.3]
Illicit substance use^d^	79 (4.3) [3.4-5.2]	1 (0.3) [0.0-0.9]	27 (3.1) [1.9-4.2]	22 (6.3) [3.7-8.8]	27 (10.8) [6.9-14.7]
Academic mobility					
Academic performance in HS	1568 (85.0) [83.4-86.7]	314 (93.5) [90.8-96.1]	760 (86.7) [84.4-88.9]	290 (81.9) [77.9-86.0]	182 (72.5) [66.9-78.1]
Graduated HS	1715 (92.9) [91.7-94.0]	318 (94.4) [91.9-96.8]	820 (93.5) [91.9-95.1]	335 (94.4) [92.0-96.8]	219 (86.6) [82.3-90.8]
Enrolled in college	849 (46.6) [44.3-48.8]	186 (55.2) [49.9-60.5]	424 (48.4) [45.1-51.7]	155 (43.7) [38.5-48.9]	84 (33.2) [27.4-39.0]

Abbreviation: ACEs, adverse childhood experiences; DV, dating violence; HS, high school; NA, not applicable; PTSD, posttraumatic stress disorder.

^a^ Valid percentage reported, in all cases missing values are less than 2.2%.

^b^ Twenty seven (1.5%) participants were missing data for the ACEs measure.

^c^ Lifetime prevalence.

^d^ Illicit substance use includes use of narcotics, stimulants, opioids, and hallucinogens.

**Table 3.** Multilevel Model Logistic Regressions Showing the Association of Outcomes and Number of ACE Exposures

Outcome	Model, No. (ICC)	1 to 3 ACEs		4 to 5 ACEs		6 or more ACEs	
		OR (95% CI)^a^	*P* value^b^	OR (95% CI)^a^	*P* value^b^	OR (95% CI)^a^	*P* value^b^
Mental health							
Depression	1798 (0.000)	2.13 (1.56-2.91)	<.001	3.09 (2.16-4.41)	<.001	4.53 (3.06-6.70)	<.001
PTSD	1802 (0.009)	3.68 (1.66-8.17)	.001	6.67 (2.94-15.13)	<.001	16.58 (7.31-37.61)	<.001
Suicide ideation	1797 (0.020)	3.73 (2.25-6.20)	<.001	5.36 (3.12-9.21)	<.001	9.71 (5.56-16.99)	<.001
Suicide attempt	1800 (0.000)	2.31 (1.30-4.08)	.004	4.09 (2.25-7.43)	<.001	9.04 (4.95-16.51)	<.001
Experiencing DV by type							
Physical	1795 (0.006)	1.13 (0.53-2.43)	.75	2.59 (1.19-5.63)	.02	4.45 (2.05-9.68)	<.001
Sexual	1805 (0.000)	2.23 (0.77-6.51)	.14	4.32 (1.44-12.94)	.009	8.53 (2.87-25.39)	<.001
Threatening	1798 (0.002)	1.90 (0.88-4.13)	.10	3.52 (1.57-7.89)	.002	6.33 (2.83-14.17)	<.001
Psychological	1810 (0.000)	1.99 (1.26-3.16)	.003	3.87 (2.72-7.63)	<.001	4.55 (2.72-7.63)	<.001
DV perpetration by type							
Physical	1796 (0.000)	2.19 (0.84-5.72)	.11	3.93 (1.47-10.52)	.007	7.66 (2.88-20.34)	<.001
Sexual^c^	1804 (0.078)	NA	NA	1.56 (0.57-4.29)	.38	4.94 (2.12-11.52)	<.001
Threatening	1796 (0.000)	2.06 (0.78-5.39)	.14	4.15 (1.55-11.10)	.005	9.80 (3.72-25.81)	<.001
Psychological	1810 (0.010)	2.28 (1.35-3.85)	.002	4.58 (2.63-7.96)	<.001	7.50 (4.24-13.24)	<.001
Other violence							
Sexual assault	1800 (0.009)	2.76 (1.28-5.92)	.009	4.84 (2.19-10.69)	<.001	13.18 (5.98-29.02)	<.001
Physical fight	1798 (0.000)	1.72 (0.79-3.74)	.17	4.55 (2.08-9.96)	<.001	7.47 (3.39-16.47)	<.001
Substance use							
Binge drinking	1786 (0.008)	1.37 (0.82-2.30)	.24	2.35 (1.34-4.10)	.003	2.79 (1.55-5.02)	.001
Marijuana use	1804 (0.007)	1.58 (1.07-2.34)	.02	2.88 (1.89-4.39)	<.001	4.00 (2.56-6.24)	<.001
Illicit substance use	1802 (0.028)	9.14 (1.23-67.84)	.03	16.88 (2.24-127.14)	.006	27.83 (3.70-209.31)	.001
Academic mobility							
Academic performance in HS	1809 (0.035)	0.57 (0.35-0.92)	.02	0.43 (0.25-0.73)	.002	0.29 (0.17-0.49)	<.001
Graduated HS	1813 (0.045)	1.09 (0.63-1.89)	.77	1.40 (0.71-2.73)	.33	0.59 (0.32-1.11)	.10
Enrolled in college	1812 (0.120)	0.99 (0.74-1.34)	.96	0.88 (0.61-1.25)	.47	0.62 (0.42-0.93)	.02

Abbreviations: ACE, adverse childhood experience; DV, dating violence; HS, high school; ICC, intraclass correlation; NA, not applicable; OR, odds ratio; PTSD, posttraumatic stress disorder.

^a^ Referent category, ACEs.

^b^ *P* values from Wald test of regression coefficient.

^c^ Referent category, ACEs and 1 to 3 ACES combined due to small cell counts.

As shown in Table 4, in our expanded measurement of ACEs, exposure to adverse neighborhood experiences was the most frequently endorsed ACE domain (815 [44.7%]), followed by experiencing discrimination (690 [37.9%]), emotional abuse as a child (532 [29.4%]), mental illness in the household as a child (489 [26.8%]), and physical abuse as a child (483 [26.7%]). As shown in eTable 2 in Supplement 1, all domains were correlated with at least one other domain. The correlations most clearly observed were between physical and emotional abuse as a child and between bullying and witnessing domestic violence (both ρ = 0.54). We next examined the independent and relative associations of individual ACE domains (ie, specific types of adversity) with selected mental health (ie, depression and PTSD) and interpersonal violence (ie, experiencing and perpetrating physical, threatening, and sexual DV) outcomes.

**Table 4.** Endorsement of ACEs

ACEs domain	No. (%)				
	Overall (n = 1824)	Experienced DV subsample (n = 197)	DV perpetration subsample (n = 144)	PTSD subsample (n = 207)	Depression subsample (n = 689)
Emotional abuse	532 (29.4)	85 (43.4)	58 (40.6)	109 (52.7)	306 (44.7)
Physical abuse	483 (26.7)	79 (40.5)	67 (47.2)	92 (44.7)	251 (36.6)
Sexual abuse	192 (10.5)	46 (23.4)	35 (24.3)	67 (32.4)	116 (16.8)
Emotional neglect	174 (9.5)	21 (10.7)	19 (13.2)	25 (12.1)	74 (10.7)
Physical neglect	190 (10.4)	28 (14.2)	22 (15.3)	29 (14.0)	63 (9.1)
Domestic violence	298 (16.4)	61 (31.0)	57 (39.6)	57 (27.5)	140 (20.4)
Substance use	429 (23.6)	75 (38.5)	60 (42.3)	91 (44.0)	209 (30.4)
Mental illness	489 (26.8)	87 (44.4)	66 (46.2)	98 (47.3)	260 (37.8)
Incarcerated	260 (14.3)	49 (25.0)	43 (30.1)	61 (29.5)	123 (17.9)
Witnessed violence	310 (17.0)	46 (23.5)	33 (23.1)	48 (23.3)	108 (15.7)
Felt discrimination	690 (37.8)	102 (51.8)	82 (56.9)	114 (55.1)	298 (43.3)
Neighborhood cohesion and safety	815 (44.7)	102 (51.8)	79 (54.9)	131 (63.3)	360 (52.2)
Bullied	267 (14.7)	47 (24.0)	33 (23.1)	41 (19.9)	112 (16.3)
Foster care	68 (3.7)	18 (9.2)	20 (14.0)	13 (6.3)	34 (5.0)

Abbreviations: ACEs, adverse childhood experiences; DV, dating violence; PTSD, Posttraumatic stress disorder.

As shown in Table 5, 10 of the 14 ACE domains were independently associated with higher odds of depression, PTSD, and DV perpetration. For experiencing DV, 11 ACE domains were independently linked. Furthermore, 8 ACE domains were consistently associated with all 4 selected outcomes (ie, emotional abuse as a child, physical abuse as a child, sexual abuse as a child, witnessing domestic violence, substance use in the household, mental illness in the household, incarceration of a household member, and experiencing discrimination). Being in foster care was associated with all outcomes except PTSD. Exposure to adverse neighborhood experiences was associated with depression and PTSD, while being bullied was associated with experiencing and perpetrating DV. Experiencing physical and emotional neglect as a child was not independently associated with any of the 4 selected outcome measures.

**Table 5.** Multilevel Logistic Regression Models of Associations of ACE Domains

ACE domain	Depression^a^			PTSD^b^			Experienced DV ^a^			DV perpetration^a^		
	No.	OR (95% CI)	*P* value	No.	OR (95% CI)	*P* value	No.	OR (95% CI)	*P* value	No.	OR (95% CI)	*P* value
**Model 1: independent associations of ACE domains**												
Emotional abuse	1782	2.90 (2.90-3.62)	<.001	1787	2.58 (1.89-3.53)	<.001	1780	1.81 (1.32-2.49)	<.001	1776	1.51 (1.05-2.19)	.03
Physical abuse	1781	2.19 (1.75-2.75)	<.001	1786	2.33 (1.70-3.20)	<.001	1779	1.91 (1.39-2.63)	<.001	1775	2.54 (1.77-3.64)	<.001
Sexual abuse	1793	2.26 (1.63-3.15)	<.001	1798	4.13 (2.85-5.98)	<.001	1792	2.64 (1.78-3.90)	<.001	1788	2.46 (1.59-3.80)	<.001
Emotional neglect	1795	1.14 (0.82-1.59)	.44	1800	1.18 (0.74-1.90)	.49	1793	1.04 (0.64-1.71)	.86	1789	1.29 (0.76-2.19)	.35
Physical neglect	1795	0.79 (0.56-1.10)	.16	1800	1.19 (0.75-1.89)	.45	1793	1.32 (0.84-2.07)	.23	1789	1.26 (0.76-2.08)	.37
Domestic violence	1784	1.64 (1.26-2.14)	<.001	1789	2.05 (1.43-2.94)	<.001	1782	2.55 (1.80-3.60)	<.001	1778	3.65 (2.51-5.31)	<.001
Substance use	1792	1.70 (1.35-2.15)	<.001	1796	2.48 (1.80-3.41)	<.001	1789	1.85 (1.34-2.56)	<.001	1785	2.21 (1.53-3.19)	<.001
Mental illness	1793	2.14 (1.71-2.69)	<.001	1798	2.18 (1.59-3.00)	<.001	1791	2.03 (1.47-2.78)	<.001	1787	2.24 (1.56-3.21)	<.001
Incarcerated	1792	1.53 (1.15-2.03)	.004	1797	2.55 (1.77-3.68)	<.001	1790	1.76 (1.21-2.55)	.003	1786	2.29 (1.53-3.42)	<.001
Witnessed violence	1794	0.95 (0.72-1.24)	.69	1799	1.68 (1.15-2.45)	<.001	1792	1.52 (1.05-2.20)	.028	1788	1.35 (0.88-2.06)	.17
Felt discrimination	1794	1.47 (1.19-1.81)	<.001	1799	2.12 (1.54-2.90)	<.001	1792	1.81 (1,32-2.47)	<.001	1788	2.01 (1.41-2.88)	<.001
Neighborhood	1795	1.54 (1.25-1.88)	<.001	1800	2.01 (1.46-2.75)	<.001	1793	1.13 (0.83-1.54)	.43	1789	1.31 (0.92-1.86)	.14
Bullied	1794	1.25 (0.95-1.66)	.12	1799	1.40 (0.94-2.08)	.10	1792	1.85 (1.28-2.67)	.001	1788	1.54 (1.01-2.36)	.05
Foster care	1791	2.03 (1.22-3.39)	.007	1796	1.93 (0.99-3.78)	.05	1789	3.12 (1.74-5.59)	<.001	1785	5.12 (2.87-9.13)	<.001
**Model 2: relative associations of ACE domains**												
No.	1761	NA	NA	1765	NA	NA	1759	NA	NA	1759	NA	NA
ICC	0.000	NA	NA	0.008	NA	NA	0.000	NA	NA	0.000	NA	NA
Emotional abuse	NA	2.17 (1.65-2.86)	<.001	NA	1.58 (1.06-2.36)	.03	NA	1.30 (0.87-1.95)	.20	NA	0.76 (0.47-1.23)	.27
Physical abuse	NA	1.12 (0.83-1.52)	.45	NA	1.04 (0.68-1.61)	.85	NA	0.93 (0.60-1.44)	.76	NA	1.38 (0.84-2.29)	.21
Sexual abuse	NA	1.39 (0.96-2.02)	.08	NA	2.37 (1.56-3.60)	<.001	NA	1.67 (1.06-2.61)	.03	NA	1.28 (0.77-2.13)	.34
Emotional neglect	NA	0.99 (0.69-1.43)	.97	NA	0.88 (0.52-1.47)	.62	NA	0.92 (0.54-1.55)	.74	NA	1.05 (0.59-1.86)	.87
Physical neglect	NA	0.62 (0.42-0.91)	.02	NA	0.83 (0.49-1.39)	.48	NA	0.87 (0.52-1.44)	.58	NA	0.77 (0.43-1.36)	.37
Domestic violence	NA	0.95 (0.68-1.33)	.76	NA	1.03 (0.66-1.63)	.89	NA	1.52 (0.98-2.37)	.06	NA	1.99 (1.22-3.24)	.006
Substance use	NA	1.04 (0.78-1.39)	.79	NA	1.31 (0.88-1.96)	.18	NA	1.14 (0.76-1.71)	.53	NA	1.32 (0.83-2.10)	.24
Mental illness	NA	1.55 (1.18-2.02)	.001	NA	1.18 (0.81-1.73)	.40	NA	1.38 (0.94-2.03)	.10	NA	1.29 (0.82-2.03)	.27
Incarcerated	NA	0.97 (0.68-1.33)	.85	NA	1.42 (0.93-2.19)	.11	NA	1.05 (0.68-1.63)	.83	NA	1.22 (0.75-1.98)	.42
Witnessed violence	NA	0.75 (0.53-1.06)	.11	NA	1.33 (0.83-2.12)	.23	NA	0.96 (0.60-1.54)	.87	NA	0.80 (0.46-1.38)	.42
Felt discrimination	NA	1.34 (1.05-1.70)	.02	NA	1.75 (1.23-2.49)	.002	NA	1.43 (1.01-2.04)	.05	NA	1.61 (1.07-2.42)	.02
Neighborhood cohesion and safety	NA	1.28 (1.03-1.59)	.03	NA	1.59 (1.13-2.24)	.008	NA	0.95 (0.68-1.34)	.78	NA	1.11 (0.75-1.64)	.61
Bullied	NA	1.39 (0.96-2.00)	.08	NA	0.91 (0.56-1.48)	.69	NA	1.40 (0.88-2.21)	.16	NA	1.04 (0.60-1.79)	.88
Foster care	NA	1.21 (0.68-2.16)	.52	NA	0.85 (0.39-1.85)	.68	NA	1.35 (0.69-2.68)	.38	NA	2.32 (1.17-4.60)	.02

Abbreviations: ACE, adverse childhood experience; DV, dating violence; ICC, intraclass correlation; PTSD, posttraumatic stress disorder

^a^ ICC estimated less than 0.000 for all models.

^b^ ICC ranged from 0.005 to 0.016.

Four ACE domains exhibited a relative association to depression, with the most prevalent being for emotional abuse as a child (OR, 2.17; 95% CI, 1.65-2.86; *P* < .001), followed by mental illness in the household (OR, 1.55; 95% CI, 1.18-2.02; *P* = .001), experiencing discrimination (OR, 1.34; 95% CI, 1.05-1.70; *P* = .02), and exposure to adverse neighborhood experiences (OR, 1.28; 95% CI, 1.03-1.59; *P* = .03). Physical neglect as a child showed a relative protective association to the incidence of depression (OR, 0.62; 95% CI, 0.42-0.91; *P* = .02). Four domains were linked to PTSD, with sexual abuse as a child exhibiting the most apparent relative association (OR, 2.37; 95% CI, 1.56-3.60; *P* < .001), followed by experiencing discrimination (OR, 1.75; 95% CI, 1.23-2.49;, *P* = .002), exposure to adverse neighborhood experiences (OR, 1.59; 95% CI, 1.13-2.24; *P* = .008), and emotional abuse as a child (OR, 1.58; 95% CI, 1.06-2.36; *P* = .03). Two domains had a relative association with emerging adult exposure to DV (sexual abuse as a child, OR, 1.67; 95% CI, 1.06- 2.61; *P* = .03, and experiencing discrimination, OR, 1.43; 95% CI, 1.01-2.04; *P* = .046) and perpetration (witnessing domestic violence, OR, 1.99; 95% CI, 1.22-3.24; *P* = .006, and experiencing discrimination (OR, 1.61; 95% CI, 1.07-2.42; *P* = .02). These findings suggest that although ACE domains frequently co-occur, several domains—particularly emotional abuse as a child, sexual abuse as a child, witnessing domestic violence, and experiencing discrimination—were more consistently associated with multiple outcomes than others.

## Discussion

In this racially and ethnically diverse cohort of emerging adults, we observed graded associations between adverse childhood experiences and mental, behavioral, and educational outcomes. ACE exposure was common, cumulative, and consistently associated with poorer functioning across domains. Together, these findings suggest that ACEs may be more informatively conceptualized, not only as cumulative burden, but as a constellation of specific adversities that may be differentially associated with risk. While the graded association between cumulative ACE exposure and adverse outcomes is well established, our findings extend this literature by demonstrating that specific forms of childhood adversity appeared to be associated with greater risk than others in emerging adulthood. In particular, emotional abuse, sexual abuse, witnessing domestic violence, and experiencing discrimination were more consistently associated with depression, PTSD, and DV than other ACE domains, even after accounting for the co-occurrence of multiple adversities. These findings suggest that conceptualizing ACEs solely as a cumulative score may obscure meaningful heterogeneity in risk across different forms of childhood adversity.

These findings are consistent with prior work connecting ACEs to adverse mental health and behavioral outcomes.^1,2,3,4,5,9,10,11,12,23,24,25,26,27,28,29,30^ Importantly, domain-specific analyses indicated that these associations were not uniform across types of adversity; rather, certain ACE domains—especially emotional abuse as a child, sexual abuse as a child, witnessing domestic violence, and experiencing discrimination—demonstrated prevalent and more consistent associations with key mental health and interpersonal violence outcomes in emerging adulthood than others. As demonstrated in previous research, bullying in childhood was associated with emerging adult DV experiences. Other notable findings included a lack of association between neglect in childhood and mental health, violence, or academic mobility outcomes. We also found significant connections between discrimination and both experiencing and perpetrating DV in emerging adulthood.

Our study extends the ACEs literature in 5 important ways. First, by using the Philadelphia ACEs measure, which includes community-level adversities (eg, racism, neighborhood safety), we assessed forms of trauma disproportionately experienced by racially and economically marginalized youths. Our findings that both conventional and community-level ACEs contributed to elevated risk underscore the importance of measuring adversity in ways that reflect the lived experiences of diverse populations. Indeed, we observed associations between experiencing discrimination and neighborhood adversity with both depression and PTSD, highlighting the mental health burden of community-level exposures. Experiencing discrimination also co-occurred with other ACE domains, suggesting that these experiences rarely occur in isolation. Together, our findings support the value of an expanded ACE framework that incorporates adverse experiences that arise in community and structural contexts (eg, neighborhood violence, discrimination) to more fully capture risk and better understand their impact on mental health.

Second, we measured outcomes during emerging adulthood—a developmental period when early adversity may exert especially potent effects on mental health, relationship functioning, and educational trajectories, and when prevention efforts may be especially impactful.^13^ Measuring ACEs close to the developmental period in which they occurred reduces this concern and allows for a clearer view of near-term impacts.^31^ These findings emphasize emerging adulthood as a critical window for intervention. Additionally, our diverse sample of emerging adults was majority (53%) noncollege attending, extending our knowledge of the affect of ACEs in the time point beyond traditionally used college samples.

Third, we examined a wide range of outcomes simultaneously, demonstrating that ACE exposure is associated with adverse outcomes across multiple domains of functioning, including mental health, substance use, interpersonal violence, and academic mobility. This broad pattern of associations is consistent with the conceptualization of ACEs as a cross-cutting risk factor that affects multiple aspects of health and functioning.^2,7^ While exposure to 1 to 3 events indicated substantial health risks across mental health, and 4 or more for substance use and interpersonal violence domains, those exposed to 6 or more ACEs demonstrated the most apparent associations with adverse health outcomes. We also move beyond the conventional ACE threshold of 4 or more exposures established in seminal research^1^ by examining graded categories, allowing for a more nuanced assessment of dose-response associations across levels of adversity.

Fourth, by examining the relative contributions of individual ACE domains (ie, specific forms of adversity), we move beyond cumulative risk models and suggest a shift in how ACEs are conceptualized—from a simple count of exposures to a construct in which specific forms of adversity appear to be associated with distinct and sometimes disproportionately greater risk. Although ACEs were highly interrelated, several domains, including emotional abuse as a child, sexual abuse as a child, witnessing domestic violence, and experiencing discrimination emerged as particularly consistent estimators of depression, PTSD, and DV, whereas others (eg, neglect as a child) were not independently associated when accounting for cooccurring adversities. These findings highlight the heterogeneity of adversity and suggest that both type and accumulation matter for understanding risk.

Finally, our findings suggest that the effects of ACEs in emerging adulthood extend beyond health to include meaningful economic consequences. The reduced academic mobility observed among participants with 6 or more ACEs may place individuals at increased risk for long-term financial instability. One plausible pathway is through mental health: ACE-associated symptoms (eg, depression) may undermine academic engagement, leading to poorer grades, reduced attendance, and decreased likelihood of college enrollment or completion.^32^ Because educational attainment is closely tied to lifetime earnings^33^ and upward mobility,^34^ supporting the academic success of young people with high ACE exposure is critical for improving long-term economic outcomes.

Given the substantial burden of poor mental health, substance use, interpersonal violence, and educational disruption in emerging adulthood, these findings highlight the need for trauma-informed prevention and early intervention. Our findings are consistent with broader evidence suggesting that routine screening for adversity in clinical and educational settings may help identify youths at heightened risk.^35^ Our findings further suggest that screening and intervention efforts may benefit from greater specificity. Because certain ACE domains (eg, emotional abuse as a child, sexual abuse as a child, and experiencing discrimination) exhibited relative associations with key selected outcomes, identifying these experiences may help prioritize individuals at highest risk and inform more tailored intervention strategies. Programs addressing shared risk and protective factors (eg, *Fourth R*^36^) may mitigate downstream effects of adversity. Because community-level adversities like experiencing discrimination and exposure to neighborhood violence also confer substantial risk, our findings, together with the broader literature, suggest that effective prevention should extend beyond the classroom, clinic, and home and include broader structural interventions that reduce childhood poverty, improve neighborhood safety, and address discrimination writ large. In addition, the prominent role of experiencing discrimination and other community-level adversities underscore the need for interventions that explicitly address structural inequities alongside individual- and family-level risk.

### Limitations

Limitations include reliance on self-reported data and the observational design of the study. Although participants were relatively close in age to the reported adversities, responses remained subject to recall bias. While the study design precludes causal inference, the temporality of adversity preceding outcomes lends plausibility to the observed associations. Although the ACE measure captured adversities occurring before age 18 years, it did not assess their timing, duration, frequency, or chronicity. Consequently, we could not examine whether the developmental timing or persistence of specific adversities associated with emerging adult outcomes. Although our diverse sample enhances generalizability, findings may not extend beyond urban US populations. ACE domain analyses were limited to selected mental health and interpersonal violence outcomes to maintain model parsimony and may not generalize to other outcomes. Because multiple related outcomes were examined, some statistically significant findings may reflect type I error despite the consistency of the overall pattern of associations. Future research should include more robust physical health outcomes.

## Conclusion

In this cohort study of emerging adults, conventional and community-level adverse childhood experiences were associated with poorer mental health, substance use, interpersonal violence, and reduced academic mobility. Importantly, specific forms of adversity were more consistently associated with adverse outcomes than others, suggesting that prevention and intervention efforts may be strengthened by targeting the most consequential domains of childhood adversity. Treating ACEs as interchangeable exposures may obscure important differences in risk, particularly for high-impact adversities such as abuse and discrimination. Comprehensive, multilevel prevention strategies that integrate trauma-informed care with structural interventions may help reduce the burden of adverse outcomes associated with childhood adversity during emerging adulthood and potentially improve longer-term health and social trajectories.

## Notes

Supplement 1. eTable 1. Coding and Prevalence of ACE Domains (Analytic Sample, n = 1850) eTable 2. Correlation Among Types of ACEs

Supplement 2. Data Sharing Statement
