# Supplementary material for: Childhood Adversity and Health in Emerging Adulthood
Source: JAMA Netw Open. 2026 Sep 24;9(9):e2635834. doi: 10.1001/jamanetworkopen.2026.35834 (PMC13613176; doi:10.1001/jamanetworkopen.2026.35834)
Supplement: Supplementary file 1 [file jamanetwopen-e2635834-s001.pdf]

Supplemental Online Content

Temple JR, Baumler E, Wood L, Taylor L. Childhood adversity and health in emerging adulthood. *JAMA Netw Open.* 2026;9(9):e2635834. doi:10.1001/jamanetworkopen.2026.35834

eTable 1. Coding and Prevalence of ACE Domains (Analytic Sample, n = 1850)

eTable 2. Correlation Among Types of ACEs

This supplemental material has been provided by the authors to give readers additional information about their work.

© 2026 Temple JR et al. *JAMA Network Open*.

eTable 1: Coding and Prevalence of ACE Domains (Analytic Sample, n = 1850)

ACE Domain	Coding	Item Threshold for Adversity	18 year incidence no – (%)[a]
**Emotional abuse[b]**			532 (29.4)
…swear at you, insult you, or put you down?	more than once, once, never	more than once	
…act in a way that made you afraid that you would be physically hurt?		more than once	
**Physical abuse[b]**			483 (26.7)
…push, grab, shove, or slap you?	more than once, once, never	more than once	
… hit you so hard that you had marks or were injured?		once	
**Sexual Abuse[c]**			192 (10.5)
…touch or fondle you in a sexual way or have you touch their body in a sexual way?	yes, no	yes	
…attempt to have or actually have any type of sexual intercourse, oral, anal or vaginal with you?		yes	
**Emotional Neglect**			174 (9.5)
There was someone in your life who helped you feel important or special.	very often true, often true, sometimes true, rarely true, never true	rarely true	
**Physical Neglect**			190 (10.4)
Your family sometimes cut the size of meals or skipped meals because there was not enough money in the budget for food.	very often true, often true, sometimes true, rarely true, never true	often true	

© 2026 Temple JR et al. *JAMA Network Open*.

**Witnessing Domestic Violence**[d]<br>…being slapped, kicked, punched, or beaten up?<br>…being hit or cut with an object, such as a stick, cane, bottle, club, knife or gun?	many times, a few times, once, never	a few times<br>once	298 (16.4)
**Household Substance Abuse**[e]<br>…was a problem drinker or alcoholic?<br>…used illegal street drugs or who abused prescription medications?	yes, no	yes<br>yes	429 (23.6)
**Household Mental Illness**[e]<br>…adult in the house was depressed or mentally ill?<br>…adult in the house was suicidal?	yes, no	yes<br>yes	489 (26.8)
**Incarcerated Household Member**<br>Did you live with anyone who served time or was sentenced to serve time in a prison, jail, or other correctional facility?	yes, no	yes	260 (14.3)
**Witnessed Violence**<br>How often, if ever, did you see or hear someone being beaten up, stabbed, or shot in real life?	many times, a few times, once, never	a few times	310 (17.0)
**Felt Discrimination**<br>How often did you feel that you were treated badly or unfairly because of your race or ethnicity?	very often true, often true, sometimes true, rarely true, never true	sometimes true	690 (37.8)
**Adverse Neighborhood Experience**<br>Did you feel safe in your neighborhood?<br>Did you feel people in your neighborhood looked out for each other, stood up for each other, and could be trusted?	All of the time, most of the time, some of the time, none of the time	some of the time<br>some of the time	815 (44.7)
**Bullied**			267 (14.6)

© 2026 Temple JR et al. *JAMA Network Open*.

How often were you bullied by a peer or classmate?	All of the time, most of the time, some of the time, none of the time	most of the time	
**Foster Care**<br>Were you ever in foster care?	yes, no	yes	68 (3.7)

[a]*Valid percentage reported, in all cases missing values <2.2%*

[b]*Stem for items "While you were growing up how often did a parent, stepparent, or another adult living in your home…"*

[c]*Stem for items "During the first 18 years of life, did an adult or older relative, family friend, or stranger who was at least five years older than yourself ever…"*

[d]*Stem for items "How often, if ever, did you see or hear in your home a parent, stepparent, or another adult who was helping to raise you…"*

[e]*Stem for items "Did you live with anyone who was…"*

© 2026 Temple JR et al. *JAMA Network Open*.

eTable 2: Correlation Among Types of ACEs

	Emotional Abuse	Physical Abuse	Sexual Abuse	Emotional Neglect	Physical Neglect	Domestic Violence	Substance Use	Mental Illness	Incar.	Witn Viol	Felt Disc.	Nbrhood	Bullied	Foster Care
Emotional Abuse	1													
Physical Abuse	.544[b]	1												
Sexual Abuse	.221[b]	.250[b]	1											
Emotional Neglect	.052[a]	.081[b]	.053[a]	1										
Physical Neglect	.047[a]	.106[b]	.088[b]	-0.019	1									
Domestic Violence	.246[b]	.433[b]	.202[b]	.049[a]	.233[b]	1								
Substance Use	.279[b]	.219[b]	.287[b]	0.041	.114[b]	.253[b]	1							
Mental Illness	.308[b]	.255[b]	.270[b]	0.022	.058[a]	.256[b]	.411[b]	1						
Incarcerated	.175[b]	.209[b]	.250[b]	.060[a]	.118[b]	.275[b]	.450[b]	.320[b]	1					
Witnessed Violence	0.008	.102[b]	.097[b]	0.028	.295[b]	.192[b]	.058[a]	.052[a]	.149[b]	1				
Felt Discrimination	.132[b]	.233[b]	.130[b]	0.004	.274[b]	.255[b]	.099[b]	.080[b]	.106[b]	.291[b]	1			
Neighborhood	.157[b]	.127[b]	.167[b]	.166[b]	-0.021	.091[b]	.153[b]	.144[b]	.130[b]	-0.031	.067[b]	1		
Bullied	0.035	.128[b]	.100[b]	.062[b]	.285[b]	.211[b]	.070[b]	.057[a]	.066[b]	.539[b]	.291[b]	-.047[a]	1	
Foster Care	.109[b]	.210[b]	.178[b]	.054[a]	.142[b]	.313[b]	.204[b]	.221[b]	.234[b]	.196[b]	.121[b]	-0.014	.213[b]	1

*a Correlation significant $p<0.05$
**b Correlation significant $p<0.01$

© 2026 Temple JR et al. *JAMA Network Open*.
