# Supplementary material for: Childhood Adversity and Health in Emerging Adulthood
Source: JAMA Netw Open. 2026 Sep 24;9(9):e2635834. doi: 10.1001/jamanetworkopen.2026.35834 (PMC13613176; doi:10.1001/jamanetworkopen.2026.35834)
Supplement: Supplementary file 2 [file jamanetwopen-e2635834-s002.pdf]

Data Sharing Statement

Temple. Childhood Adversity and Health in Emerging Adulthood. *JAMA Netw Open*. Published September 24, 2026. doi:10.1001/jamanetworkopen.2026.35834

Data
Data available: Yes
Data types: Deidentified participant data
How to access data: https://dash.nichd.nih.gov
When available: beginning date: 08-01-2027

Supporting Documents
Document types: None

Additional Information
Who can access the data: Researchers whose proposed use of the data has been approved
Types of analyses: For a specified purpose.
Mechanisms of data availability: After approval and with a signed data access agreement.
